# Cloning and Expression Analysis of *ATG8* (Autophagy-Related 8) Gene Family in Solanaceae

**DOI:** 10.3390/plants13202924

**Published:** 2024-10-18

**Authors:** Yahan Chen, Yunshuang Lu, Shibo Dong, Chengde Yang, Shunyi Yang

**Affiliations:** College of Plant Protection, Gansu Agricultural University, Lanzhou 730070, China; luyunshuang6@163.com (Y.L.); 18894545570@163.com (S.D.); yangcd@gsau.edn.cn (C.Y.); yangshy@gsau.edu.cn (S.Y.)

**Keywords:** *ATG8*, autophagy, gene family evolution, bioinformatics analysis, osthole

## Abstract

The autophagy-related gene family *ATG8* (Autophagy-related 8) plays an important role in plant growth, development, and stress response. In this study, 15 *ATG8* gene family sequences were amplified from Solanaceae, namely tobacco, tomato, and pepper, using RT-PCR to evaluate their basic properties, protein structure, and function, as well as the role of *ATG8* in autophagy. The physicochemical properties, the predicted secondary and tertiary protein structures, subcellular localisation, gene structures, conserved motifs, and phylogenetic relationships of the *ATG8* genes were analysed using bioinformatic techniques, and their expression patterns under sericin-induced plant disease resistance were investigated by RT-qPCR. The lengths of these proteins ranged from 79 to 120 aa, while their predicted molecular weights and isoelectric points (*PI*) ranged from 9283.62 to 13,778.74 and 6.32 to 11.44, respectively. The majority of the proteins were localised in the nucleus or chloroplasts. Conserved protein motifs and various cis-regulatory elements in the protein, with a wide range of related functions, were identified. The *ATG8* gene family members showed expression changes after treatment with osthole, which induces disease resistance in tobacco, tomato, and pepper. These findings provide a foundation for further analyses of the *ATG8* gene family in Solanaceae and the mechanism underlying the response to adverse conditions.

## 1. Introduction

Autophagy is a conserved mechanism for the degradation of intracellular components in eukaryotes, whereby functionally impaired proteins, protein complexes, and organelles are translocated to the vacuoles of plant cells or the lysosomes of animal cells for degradation and recycling by hydrolytic enzymes [1,2]. Plant autophagy is divided into macroautophagy, microautophagy, and mega-autophagy. The term autophagy is commonly used to refer to macroautophagy, in which contents are surrounded by double-membrane autophagosomes and transported to the vacuole, where they fuse with the vacuolar membrane to form single-membrane autophagic bodies and are degraded by hydrolytic enzymes [3,4]. In plants, there is a relationship between autophagy and resistance; in particular, autophagy is involved in the responses to various environmental stresses such as nutrient deficiency, drought, salinity, and high temperature. This suggests that autophagy has the ability to improve crop response to environmental stress [5,6]. Furthermore, autophagy dysfunction is associated with the development of various diseases and viral infections, such as *Sclerotinia sclerotiorum*, tobacco mosaic virus (TMV), cotton leaf curl Multan virus (CLCuMuV), and tomato yellow leaf curl virus (TYLCV). Autophagy can either inhibit or promote viral replication, depending on the virus type, thus playing a key role in regulating cell survival [7,8].

Autophagy-related genes (*ATG*) refer to genes involved in the process of autophagy. Generally, multiple *ATGs* are jointly involved in autophagy, and more than 40 *ATGs* with different functions have been identified in eukaryotes [1,9,10]. Among them, *ATG8* is a critical gene involved in the formation, elongation, and fusion of autophagic bodies [11,12,13]. Upon the activation of cellular autophagy, *ATG8* covalently modifies the inner and outer membranes of autophagosomes and binds to phosphatidylethanolamine (*PE*) to produce membrane-bound ATG8-phosphatidylethanolamine (*ATG8-PE*) conjugates, which are then localised to the membrane structures of autophagosomes and autolysosomes. Therefore, ATG8 proteins are commonly used to monitor the induction of cellular autophagy and its progression [14,15,16].

Osthole, 7-methoxy-8-(3-methylbut-2-enyl) chromen-2-one, is a natural secondary metabolite derived from the mature fruits of plants in the families Apiaceae and Rutaceae, with high concentrations in the traditional Chinese medicine *Fructus Cnidii*. Studies have shown that osthole has antibacterial, insecticidal, and antiviral activities, including against *Escherichia coli*, *Fusarium oxysporum*, *Rhizoctonia solani*, *Macrophoma kawatsukai*, *Fusarium graminearum*, and TMV [17,18,19]. However, relatively little is known about its antiviral activity [20,21]. Osthole can induce disease resistance and anti-TMV activity in tobacco plants. Furthermore, increases in autophagosomes and autophagic bodies related to autophagy in tobacco are associated with the upregulation of *ATGs* (*ATG4*, *ATG5*, *ATG6*, *ATG7*, *ATG8*, and *ATG18*), particularly *ATG8*. In view of these findings, we chose to explore the *ATG8* gene family in greater depth.

Given the crucial role of the *ATG8* family in growth, development, nutrient cycling, and the stress response in plants, we employed reverse transcription (RT)-PCR to identify and clone *ATG8* gene family members in tobacco (*Nicotiana benthamiana*), tomato (*Solanum lycopersicum*), and pepper (*Capsicum annuum*) in order to then predict their gene structures, chromosomal distribution, and conserved motifs. Pepper and tomato leaves were treated with osthole and then inoculated with TMV, while tobacco leaves sprayed with osthole were treated with the autophagy inhibitor 3-methyladenine (3-MA), followed by inoculation with TMV. Osthole-induced anti-TMV activity was evaluated by real-time fluorescence quantitative PCR. These findings provide a foundation for further analyses of the biological functions of the *ATG8* gene family in the stress responses, growth, and development in Solanaceae, providing a guide for the application of *ATG8* in the breeding of stress-resistant tobacco, tomato, and pepper plant varieties.

## 2. Results

### 2.1. Cloning of the ATG8 Gene Family

Using the cDNA obtained by the reverse transcription of the total RNA extracted from the leaves of tobacco, pepper, and tomato plants as templates, RT-PCR was performed using specific primers designed using Primer 5.0. The resulting products were detected by 2.0% agarose gel electrophoresis. The sizes were consistent with the expected results.

### 2.2. Bioinformatics Analysis of the ATG8 Gene Family

#### 2.2.1. Prediction of the Physicochemical Properties and Subcellular Localisation of ATG8 Proteins

The 15 proteins encoded by the *ATG8* gene identified in leaf samples from three species were 79–120 aa in length, with predicted protein molecular weights (M_r_) of 9283.62–13,778.74 and pI values of 6.32–11.44. The protein instability index was >40 for all sequences, with the exception of *ATG8b1-Nt* (37.53), *ATG8d1-Nt* (36.65), and *ATG8d2-Nt* (35.06), suggesting that most of the identified ATG8 proteins were unstable. The grand average of hydropathy values for these proteins was negative, indicating that they were all hydrophilic (Table 1). A subcellular localisation analysis showed that most *ATG8* genes were found in the nucleus, while a small number were present in chloroplasts. These results suggest that the *ATG8* gene in Solanaceae mainly exerts functions related to particular biological processes in the nucleus.

#### 2.2.2. Secondary and Tertiary Structure Prediction of ATG8 Proteins

**Figure 1:**
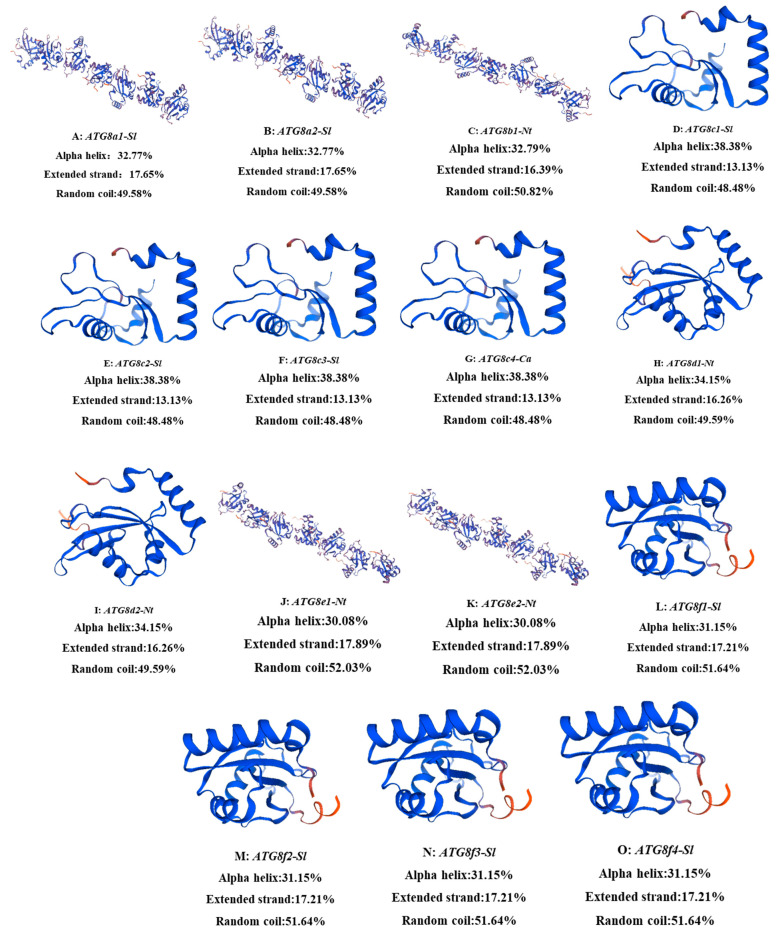
Prediction of tertiary structures of ATG8 gene family proteins (A–O).

The protein secondary structure was predicted using the SOPMA online tool, revealing that random coils were most common in the ATG8 proteins (accounting for 48.48–52.03% of structural elements), followed by α-helices (31.15–38.38%) and extended strands (13.13–17.89%) (Figure 1). SWISS MODEL was used to predict the tertiary structure of the 15 ATG8 target fragment proteins in this study, and the model diagram is shown in Figure 1.

#### 2.2.3. Analysis of ATG8 Protein Phosphorylation Sites, Transmembrane Structures, Signal Peptides, and Conformational Plausibility

Netphos3.1 Server was used to predict the protein phosphorylation sites of the proteins encoded by the *ATG8* gene family. Serine phosphorylation sites were the most common (4–15 sites), followed by threonine phosphorylation sites (0–9), while tyrosine phosphorylation sites were relatively rare (Table 2). Protein functions may be associated with phosphorylation. The prediction results obtained using the TMHMM online tool showed that none of the proteins had transmembrane regions. SignalP was also used to predict signal peptides, showing that none of the sequences in this study had signal peptide regions. In addition, the conformational plausibility of *ATG8* gene family proteins was evaluated using Ramachandran plots. The amino acids were found to be located in allowed regions, with the exception of *ATG8f2-Sl*, in which 2.9% of amino acid residues were located in disallowed regions (Table 3). The percentage of amino acids in additional allowed regions exceeded 95% for all sequences obtained in this study. Therefore, the majority of the spatial models obtained from ATG8 homology modelling were reliable and plausible.

#### 2.2.4. Analysis of ATG8 Gene Structures and Chromosomal Localisation

**Figure 2:**
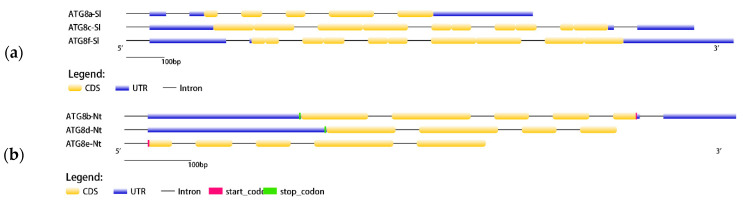
Visual analysis of *ATG8* gene structures: (**a**) Tomato gene structure; (**b**) Tobacco gene structure.

**Figure 3:**
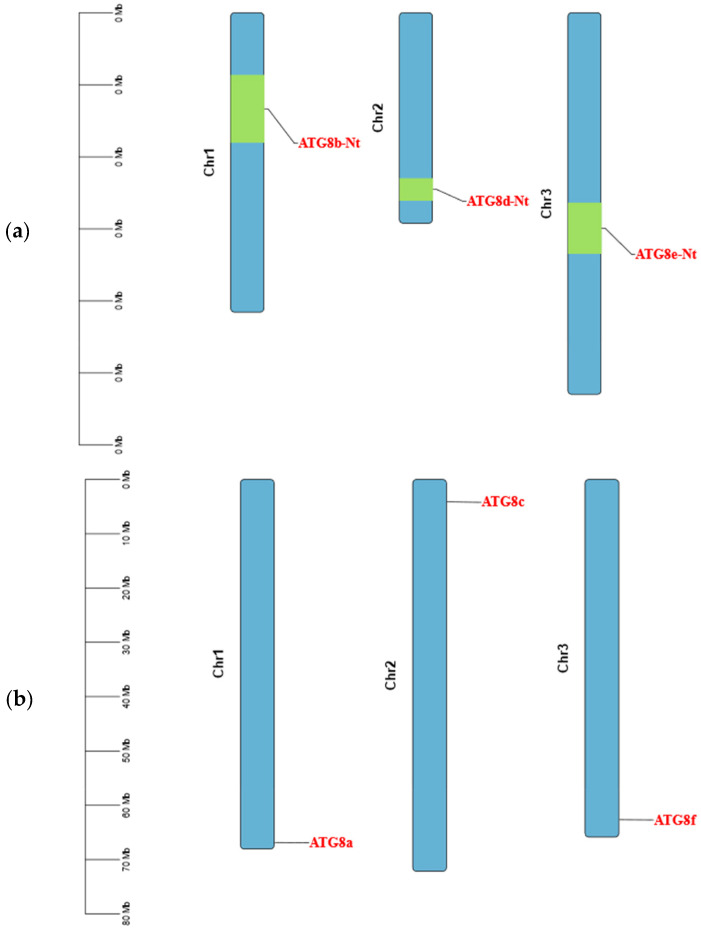
*ATG8* chromosome mapping. Localisation of *ATG8* on (**a**) tomato and (**b**) tobacco chromosomes.

Detailed bioinformatics analyses were performed to evaluate *ATG8* in tomato and tobacco plants. To clarify the composition of the *ATG8* gene family, the gene structures of the tomato and tobacco genome sequences were visualised and analysed (Figure 2). The gene structures of individual *ATG8* gene family members in both tobacco and tomato plants varied considerably, with different sequence lengths and motifs. The gene structure of the *ATG8* gene family in tomato included coding sequences (CDS), untranslated regions (UTRs), and intronic regions, with high proportions of sites classified as CDS and intronic regions. The *ATG8* gene family in tobacco showed a rich variety of gene structures, including CDS, UTRs, and introns. The *ATG8* genes in tobacco plants had higher proportions of CDS, introns, and UTRs and a greater variety of regions than those in tomato plants. There was also some variation in the structure of *ATG8* genes among subfamilies. The chromosomal localisation of the *ATG8* genes in the tomato and tobacco plants was evaluated using TBtools. As shown in Figure 3, different *ATG8* genes in tomato and tobacco were distributed on different chromosomes, with one *ATG8* gene on each chromosome.

#### 2.2.5. Analysis of Conserved Motifs and Cis-Acting Elements

**Figure 4:**
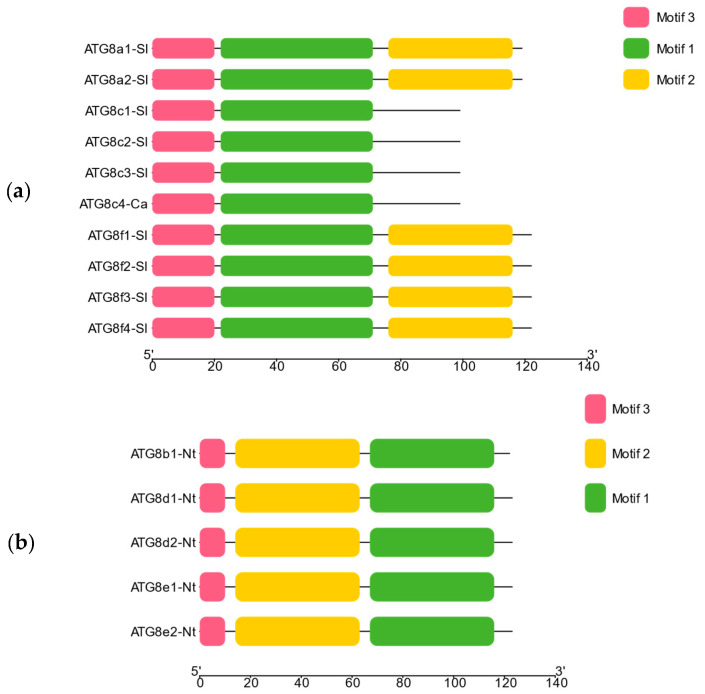
*ATG8* conserved motif analysis: (**a**) Conserved motif analysis of *ATG8* in tomato. (**b**) Conserved motif analysis of *ATG8* in tobacco.

To evaluate the evolution of the *ATG8* gene family, MEME online software was used to analyse conserved motifs in the tobacco and tomato plants. As shown in Figure 4, ten motifs with high levels of conservation were obtained in tomato (Motif1–Motif10), while four conserved motifs were identified in tobacco (Motif1–Motif4). Overall, the conserved motifs of the *ATG8* gene family varied across subfamilies, indicative of differences in the conservation and function of *ATG8* genes among species.

**Figure 5:**
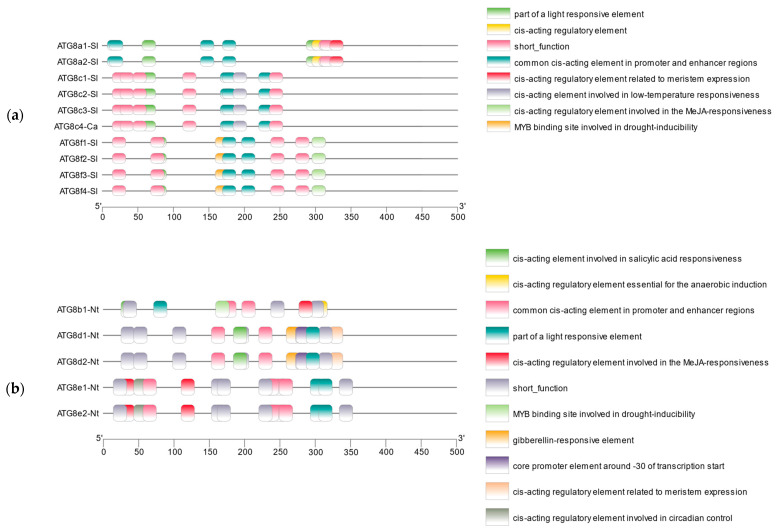
Analysis of *ATG8* cis-regulatory elements: (**a**) Analysis of cis-regulatory elements of *ATG8* in tomato. (**b**) Analysis of cis-regulatory elements of *ATG8* protein in tobacco.

The cis-acting elements of the *ATG8* gene family in the tomato and tobacco plants were predicted and visualised using PLANTCARE combined with TBtools. Various cis-acting elements closely related to the phytohormone response, light response, tissue-specific expression, and biotic or abiotic stress were distributed in tomato and tobacco (Figure 5), with particularly varied and numerous cis-acting elements related to tissue-specific expression and biotic or abiotic stress. This result explains the relationship between *ATG8* and autophagy.

#### 2.2.6. Phylogenetic Analysis of ATG8 Gene Family

**Figure 6:**
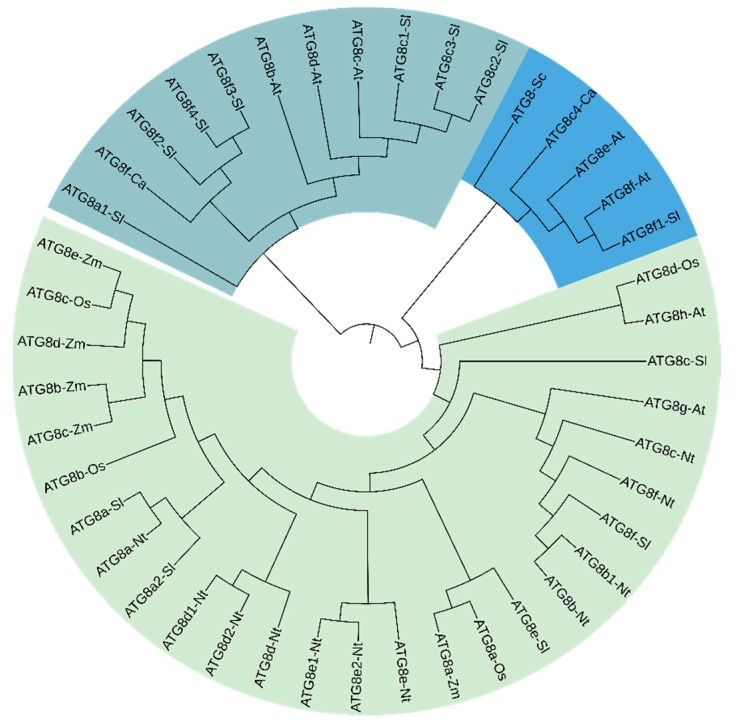
Phylogenetic analysis of ATG8 proteins.

Multiple sequence alignment was used to generate 15 *ATG8* gene sequences from tobacco, tomato, and pepper, along with a number of previously reported *ATG8* gene sequences from tobacco, pepper, tomato, yeast, *Arabidopsis thaliana*, rice, and maize. As shown in Figure 6, the resulting phylogenetic tree was divided into two main sub-branches, and the 15 *ATG8* gene sequences obtained in this study were assigned to the two sub-branches. Moreover, the *ATG8* genes in this study were found to form a cluster with those from different species. These findings indicate that there was some divergence among tobacco, tomato, and pepper. The tobacco ATG8 obtained in this study formed a cluster with ATG8 in maize and rice, suggesting they are closely related. Most tomato *ATG8* genes obtained in this study were assigned to the same branch and formed a cluster with the *ATG8* gene sequences in *A. thaliana*, suggesting that these proteins had high similarity and homology.

#### 2.2.7. Synteny Analysis

**Figure 7:**
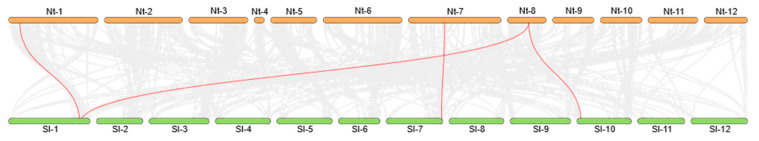
Synteny analysis of *ATG8* genes in tomato and tobacco.

A synteny analysis of the *ATG8* gene family in tobacco and tomato was performed using TBtools combined with the annotation files for tobacco and tomato. There were only four directly orthologous *ATG8* gene pairs in tobacco and tomato (Figure 7), consistent with the evolutionary divergence of the *ATG8* gene family in tobacco and tomato.

### 2.3. Analysis of ATG8 Expression in Osthole-Induced Disease Resistance in Pepper and Tomato

**Figure 8:**
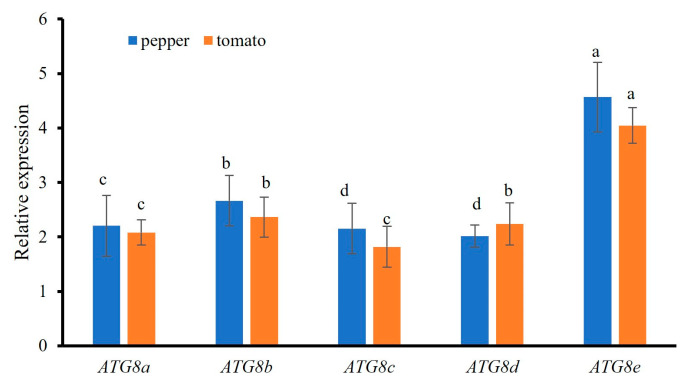
Quantitative analysis of the expression of *ATG8* genes in pepper and tomato treated with sericin. Different lowercase letters indicate significant differences (*p* < 0.05).

RT-qPCR analyses of pepper leaves treated with the preventive agent osthole were performed to evaluate *ATG8* expression. As shown in Figure 8, all *ATG8* genes were upregulated in response to osthole. In particular, *ATG8a*, *ATG8b*, *ATG8c*, *ATG8d*, and *ATG8e* were upregulated by 2.20-, 2.66-, 2.15-, 2.00-, and 4.56-fold, respectively. *ATG8e* was significantly upregulated, while the changes in *ATG8c* and *ATG8d* were not significant.

RT-qPCR was also used to evaluate *ATG8* expression in tomato leaves treated with the preventive agent osthole. All ATG8 genes were upregulated in response to osthole. In particular, *ATG8a*, *ATG8b*, *ATG8c*, *ATG8d*, and *ATG8e* were upregulated by 2.08-, 2.36-, 1.81-, 2.23-, and 4.04-fold, respectively. *ATG8e* was upregulated significantly, while the change in *ATG8d* was not significant.

### 2.4. Effect of the Autophagy Inhibitor 3-MA on ATG8 Gene Family Expression

**Figure 9:**
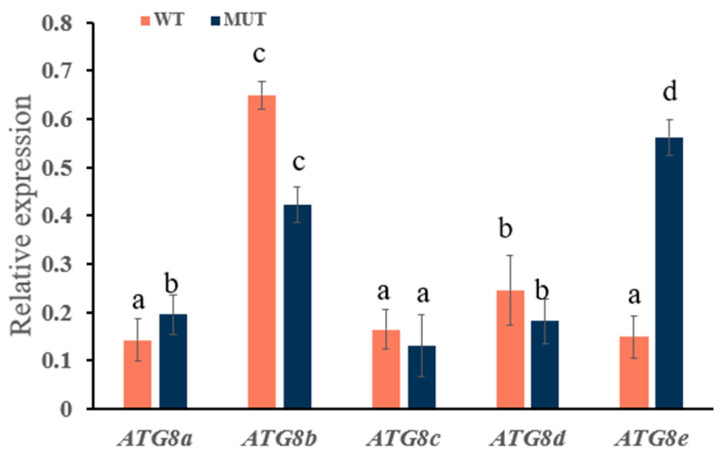
Quantitative analysis of *ATG8* gene expression levels in *N. benthamiana* leaves treated with 3-MA. Different lowercase letters indicate significant differences (*p* < 0.05).

Wild-type and *ATG8f* mutant *N. benthamiana* plants were treated with 3-MA, followed by treatment with the preventive agent osthole. According to RT-qPCR, the *ATG8* genes were downregulated in wild-type tobacco. As shown in Figure 9, in particular, *ATG8a*, *ATG8b*, *ATG8c*, *ATG8d*, and *ATG8e* were downregulated by 1.79-, 1.85-, 2.27-, 2.50-, and 1.23-fold, respectively; *ATG8c* and *ATG8d* showed the most significant decreases, while the change in *ATG8e* was not significant.

In mutant tobacco, the *ATG8* genes were all downregulated: *ATG8a*, *ATG8b*, *ATG8c*, *ATG8d*, and *ATG8e* were downregulated by 1.96-, 2.94-, 2.85-, 1.49-, and 1.79-fold, respectively. *ATG8b* and *ATG8c* were significantly downregulated, whereas *ATG8d* was not significantly downregulated.

## 3. Discussion

*ATG* genes were first identified in yeast, with 41 *ATGs* having been reported in yeast. However, *ATGs* have also been identified in plants such as *A. thaliana*, tomato, rice, and maize. Furthermore, different gene families can regulate different functions. For example, *ATG8* overexpression in *A. thaliana* can improve the N-remobilisation efficiency and promote seed filling [22]. In rice, *ATG6* is associated with the stress response (e.g., heat, cold, and drought), while in broad beans *ATGs* are known to contribute to the development of drought tolerance [23,24,25,26]. The results of previous studies are similar to those of the present study, in which an osthole-induced increase in the expression of *ATG8* genes was observed, resulting in an increased process of autophagosome formation. However, further in-depth analyses of the specific functions of the *ATG8* gene family are needed. In this study, 15 target gene sequences of the *ATG8* gene family (*ATG8a*–*f*) were cloned from three species of Solanaceae, namely tobacco, tomato, and pepper. The translated protein sequences were aligned with those of other species for phylogenetic analysis, revealing that these proteins were closely related. Furthermore, the ATG8 proteins of different species were located on different sub-branches, while those within a species were clustered together. In general, ATG8 proteins on the same branch shared similar structural and functional domains, implying functional similarities. In addition, by analysing the physicochemical properties, we concluded that ATG8 proteins in plants belonging to Solanaceae are hydrophilic and structurally unstable. Additionally, the predicted secondary and tertiary structures of the protein sequences provide a foundation for further research on the functions of ATGs and their mechanisms of action in Solanaceae.

The *ATG8* genes identified in this study tended to exhibit conserved structures and motifs. Ten highly conserved motifs (Motif1–Motif10) were found in tomato, and four highly conserved motifs (Motif1–Motif4) were found in tobacco. These results indicate that conserved motifs had a high degree of intra-specific similarity and high inter-specific variability. Furthermore, *ATG8* genes in both tomato and tobacco contained various cis-acting elements in the promoter regions. In particular, CAAT-box associated with tissue-specific expression and MYB-binding sites associated with stress responses were found to be abundant, further suggesting that *ATG8* genes are involved in the autophagy process in plants. Previous studies have found that osthole induces resistance to TMV. Thus, RT-qPCR was performed to explore the expression of the *ATG8* gene family after treatment with osthole. After the tomato and pepper plants were treated with osthole, the *ATG8* gene family members were found to be significantly upregulated, and resistance was enhanced. Wild-type and mutant tobacco plants were sprayed with the autophagy inhibitor 3-MA, followed by treatment with osthole. The RT-qPCR results indicated that *ATG8* gene family members were downregulated, and resistance in both wild-type and mutant tobacco decreased. These findings suggest that autophagy was associated with the expression of the *ATG8* gene family. Li et al. [27] treated plant leaves with autophagy inhibitors to induce the over-accumulation of starch, with small starch granules outside the chloroplasts. Treatment with autophagy inhibitors reduced the number of starch granules in the vacuoles, providing insights into the physiological function of plant autophagy. There is evidence to suggest that plant autophagy is caused by a large increase in ROS, which can serve as a signalling molecule to directly or indirectly activate the expression of resistance and defence genes, in turn promoting autophagy. However, the precise mechanisms are unclear and should be a focus of further investigations [28]. Taken together, these findings provide a foundation for further research on the function of *ATG8* genes in Solanaceae and their mechanism of action in the plant antiviral response and the growth and development of Solanaceae.

## 4. Materials and Methods

### 4.1. Test Strains and Vectors

*Escherichia coli* DH5α and the cloning vector pClone007 were purchased from Tsingke Biotechnology Co., Ltd. (Xi’an, China).

### 4.2. Test Plants

Wild-type and *ATG8f* mutant *N. benthamiana* were grown in a climate chamber at a constant temperature of 22 ± 3 °C with an alternating light (16 h) and dark (8 h) cycle for 3–6 weeks before use. Pepper (“Luosijiao”) and tomato (“Dongfen 108”) seedlings were purchased from Shandong Shouguang Ruiheng Seed Industry and cultivated at 22 °C until the seedlings were about 4 weeks old.

### 4.3. Reagents and Culture Media

The following kits and reagents were used: osthole (Shanghai Yuanye Biotechnology Co., Ltd.) (Shanghai, China), TMV (stored in the laboratory in a −80 °C freezer), TRIzol reagent (Tsingke Biotechnology Co., Ltd.) (Xi’an, China), FastKing gDNA Dispelling RT SuperMix Reverse Transcription Kit (TIANGEN Biotech Co., Ltd.) (Beijing, China), SYBR^®^ Green Pro Taq HS Premixed qPCR Kit (with Rox) (Hunan Ecorui Biological Engineering Co., Ltd.) (Changsha, China), 2000 DNA Marker (Biomed Co., Ltd.) (Shanghai, China), Plasmid Miniprep Kit (TIANGEN Biotech Co., Ltd.) (Beijing, China), DNA Gel Recovery Kit (Tsingke Biotechnology Co., Ltd.) (Xi’an, China), TaKaRa PrimeScript II 1st Stand cDNA Synthesis Kit (Tsingke Biotechnology Co., Ltd.) (Xi’an, China), PrimeSTAR^®^ GXL DNA Polymerase (Tsingke Biotechnology Co., Ltd.) (Xi’an, China), TaKaRa Ex Taq^®^ DNA Polymerase and restriction endonucleases Kpn I and Xba I (TaKaRa Bio Inc.) (Beijing, China), LB (solid/liquid) medium, and 50 μg/mL kanamycin (Kan) and ampicillin (Amp).

### 4.4. Cloning of Target Genes

#### 4.4.1. Total RNA Extraction

The total RNA of the pepper, tomato, and tobacco leaf samples were extracted using the TRIzol method and dissolved in 40 μL of nuclease-free water. After the RNA concentration was determined using the Unano-2000 Microvolume Nucleic Acid Analyser (Thermo Fisher Scientific) (Waltham, MA, USA), RNA integrity was detected by agarose gel electrophoresis. The total RNAs extracted from the samples were stored in a −80 °C freezer for later use.

#### 4.4.2. cDNA Synthesis

The total RNA extracted from the tomato, pepper, and tobacco leaves were used as templates for cDNA synthesis. RT was carried out according to the instructions provided with the TaKaRa PrimeScript II 1st Strand cDNA Synthesis Kit, and the resulting cDNA was stored in a −20 °C freezer with 10 μL of deuterium-depleted water.

#### 4.4.3. RT-PCR

Using the full-length sequences of *ATG8a*, *ATG8b*, *ATG8c*, *ATG8d*, and *ATG8e* in the GenBank nucleic acid sequence database as reference sequences, specific primers were designed using Primer 5.0, as shown in Table 4. The PCR system was as follows: cDNA (2.0 μL), 5× PS GXL Buffer (5 μL), dNTP Mix (2.0 μL), upstream primer (1.0 μL), downstream primer (1.0 μL), and PS GXL DNA Polymerase (0.5 μL), with sterile water up to 25 μL. The PCR settings were as follows: 98 °C for 10 s, 55 °C or 60 °C for 15 s, and 68 °C for 2 min, for 35 cycles. The amplification products were subjected to electrophoresis using 2.0% agarose gel stained with nucleic acid dye. The results were observed, and images were obtained using a gel imager. Bands containing the target sequences were excised and recovered. The purified products were ligated into the pMD19-T vector. After incubating at room temperature (22–23 °C) for 5 min, the ligation product was transformed into *Escherichia coli* DH5α-competent cells. Single clones were selected for shaking culture at 37 °C, and the bacterial solution was used as a template for PCR. Three bacterial solutions with positive results were screened and sent to Biomed Co., Ltd. (Shanghai, China) for sequencing.

### 4.5. Bioinformatics Analysis

All *ATG8* opening reading frames (ORFs) were translated into amino acid sequences using SnapGene 4.3.6. The number of amino acid residues, theoretical isoelectric point (*PI*), molecular weight, and hydrophilicity or hydrophobicity of the *ATG8* family members were analysed online using the ExPASy website (https://web.expasy.org/protscale/). The protein secondary and tertiary structures were predicted using NPSA (https://npsa-prabi.ibcp.fr/cgi-bin/npsa_automat.pl?page=npsa_sopma.html) and SWISS-MODEL (https://swissmodel.expasy.org/), respectively. Subcellular localisation was predicted using Cell-PLoc (http://www.csbio.sjtu.edu.cn/bioinf/Cell-PLoc-2/). Possible phosphorylation sites were predicted using the NetPhos 3.1 Server (http://www.cbs.dtu.dk/services/NetPhos/). The transmembrane structure of the ATG8 protein was predicted using the TMHM (https://services.healthtech.dtu.dk/services/TMHMM-2.0/) online tool [29,30,31]. The ATG8 signal peptides were predicted using SignalP (https://services.healthtech.dtu.dk/services/SignalP-5.0/). The homology modelling results for ATG8 proteins were evaluated using SAVES (https://saves.mbi.ucla.edu/) [32].

Published sequences of the *ATG8* family members were downloaded from the NCBI (https://www.ncbi.nlm.nih.gov/) database for tobacco, tomato, pepper, *Saccharomyces*, *A. thaliana*, *Oryza sativa*, and maize (*Zea mays*). A sequence alignment was generated using ClustalW. The neighbour-joining (NJ) method was used to construct a phylogenetic tree, with 1000 bootstrap replicates and a phylogenetic tree display threshold of 50% [33,34,35,36]. The resulting phylogenetic tree was manipulated for visualisation using iTOL (https://itol.embl.de/).

TBtools software (2024.1.11) was employed in conjunction with the GFF files of the tobacco and tomato plants for visualisation and synteny analyses of the structure and chromosomal location of the *ATG8* gene family members. The conserved motifs were analysed using the online software MEME (http://meme-suite.org/). Promoter cis-acting elements were predicted using PLANTCARE (https://bioinformatics.psb.ugent.be/webtools/plantcare/html/). Dates of all website visits accessed on 24 September 2024.

### 4.6. ATG8 Expression During Osthole-Induced Disease Resistance in Pepper and Tomato

Four-week-old pepper and tomato seedlings of uniform size and morphology were selected, and their leaves were sprayed with 1 mg/mL osthole. For the control, solvent was sprayed on pepper and tomato leaves. After one day, rub-inoculation was performed using 40 µL of TMV at a concentration of 10 mg/mL. The pepper and tomato leaves were collected on the seventh day after inoculation, flash-frozen in liquid nitrogen, and stored in a −80 °C freezer for subsequent experiments.

RNA was extracted from the pepper and tomato plants using the TRIzol method. Following the instructions provided with the FastKing gDNA Dispelling RT SuperMix (TIANGEN) Kit, total plant RNA was used as a template. The reaction system was as follows: total RNA (2 µL), 5× FastKing-RT SuperMix (4 μL), and RNase-Free ddH_2_O (12 μL). The procedure was as follows: genomic DNA removal and reverse transcription at 42 °C for 15 min and enzyme deactivation using the delta method [37] at 95 °C for 3 min. The resulting cDNA was diluted with 10 μL of DDW and stored in a −20 °C freezer.

Following the instructions provided with the SYBR^®^ GreenPremix Pro Taq HS qPCR Kit (Rox Plus) (Accurate Biology Co., Ltd., Changsha, China), the cDNA was used as a template, using the actin gene in tobacco as an internal control, for real-time fluorescence quantitative PCR using the primer pairs designed based on the *ATG8* gene sequence (Table 5). Four technical replicates and three biological replicates were evaluated for each sample. The qPCR system was as follows: 2× SYBR^®^ Green Pro Taq HS Premix (ROX Plus) (10 μL), cDNA (150 ng), Primer F (10 µM, 0.4 μL), Primer R (10 µM, 0.4 μL), and RNase-free water (8.2 μL). The reaction conditions were as follows: 95 °C for 30 s, 95 °C for 5 s, and 59 °C for 30 s (cyclic denaturation for 40 cycles), followed by 95 °C for 15 s, 60 °C for 1 min, and 95 °C for 15 s. The relative expression was calculated using the 2^−ΔΔCt^ method, with the Ct values in the control group set to 1.

### 4.7. Effect of the Autophagy Inhibitor 3-MA on ATG8 Gene Expression

Wild-type and *ATG8f* mutant *N. benthamiana* were grown in an artificial climate-controlled chamber until the four-leaf stage. Then, osthole was sprayed on the leaves of both types of tobacco, and the autophagy inhibitor 3-MA was sprayed onto the leaves one day later. The solvent of 3-MA was used as the control, and the experiment was performed in triplicate. One day later, TMV was inoculated on tobacco leaves sprayed with 3-MA or the 3-MA solvent, with phosphate buffer (PB) as the control; the experiment was performed in triplicate. Leaves were collected after the third day, flash-frozen in liquid nitrogen, and stored in a −80 °C freezer. Leaf RNA was extracted, and RT-q PCR was carried out, as described above.

### 4.8. Statistical Analysis

SPSS Statistics 26 was used to perform the statistical analysis. The significance of the statistical differences between the three means was determined using Duncan’s new complex range method at the 5% level.

## Figures and Tables

**Table 1:** Physicochemical properties of ATG8 proteins.

Gene	Protein Length (KDa)	Molecular Weight (M_r_/10^3^)	Theoretical pI	Instability Index	Aliphatic Index	Grand Average of Hydropathy	Subcellular Localisation
*ATG8a1-Sl*	119	13,744.94	8.78	40.52	84.45	−0.361	Nucleus
*ATG8a2-Sl*	119	13,744.94	8.78	40.52	84.45	−0.361	Nucleus
*ATG8b1-Nt*	122	14,031.14	6.61	32.69	92.70	−0.418	Chloroplast
*ATG8c1-Sl*	99	11,428.18	7.93	44.53	93.54	−0.415	Nucleus
*ATG8c2-Sl*	99	11,428.18	7.93	44.53	93.54	−0.415	Nucleus
*ATG8c3-Sl*	99	11,428.18	7.93	44.53	93.54	−0.415	Nucleus
*ATG8c4-Ca*	99	11,428.18	7.93	44.53	93.54	−0.415	Nucleus
*ATG8d1-Nt*	123	14,109.19	6.61	35.45	86.42	−0.385	Cytoplasm
*ATG8d2-Nt*	123	14,109.19	6.61	35.45	86.42	−0.385	Plasma membrane
*ATG8e1-Nt*	123	14,107.26	6.61	43.04	90.41	−0.335	Chloroplast
*ATG8e2-Nt*	123	14,107.26	6.61	43.04	90.41	−0.335	Chloroplast
*ATG8f1-Sl*	122	13,930.08	7.85	35.19	94.34	−0.331	Mitochondria Peroxisome
*ATG8f2-Sl*	122	13,930.08	7.85	35.19	94.34	−0.331	Nucleus
*ATG8f3-Sl*	122	13,930.08	7.85	35.19	94.34	−0.331	Nucleus
*ATG8f4-Sl*	122	13,930.08	7.85	35.19	94.34	−0.331	Nucleus

Note: Mr is the relative molecular weight.

**Table 2:** Prediction of phosphorylation sites of proteins encoded by *ATG8* gene family.

Gene	Serine	Threonine	Tyrosine
*ATG8a1-Sl*	4	1	1
*ATG8a2-Sl*	4	3	0
*ATG8b1-Nt*	4	1	0
*ATG8c1-Sl*	8	3	0
*ATG8c2-Sl*	8	3	0
*ATG8c3-Sl*	8	3	0
*ATG8c4-Ca*	15	3	0
*ATG8d1-Nt*	8	2	0
*ATG8d2-Nt*	7	3	0
*ATG8e1-Nt*	6	1	1
*ATG8e2-Nt*	5	2	1
*ATG8f1-Sl*	10	9	0
*ATG8f2-Sl*	11	0	0
*ATG8f3-Sl*	9	0	0
*ATG8f4-Sl*	9	0	0

**Table 3:** Protein homology modelling of the *ATG8* gene family.

Gene	Most Favoured Regions (%)	Additional Allowed Regions (%)	Generously Allowed Regions (%)	Disallowed Regions (%)
*ATG8a1-Sl*	88.0	12.0	-	-
*ATG8a2-Sl*	88.0	12.0	-	-
*ATG8b1-Nt*	95.5	4.5	-	-
*ATG8c1-Sl*	55.0	40.0	5.0	-
*ATG8c2-Sl*	55.0	40.0	5.0	-
*ATG8c3-Sl*	55.0	40.0	5.0	-
*ATG8c4-Ca*	92.7	2.8	-	-
*ATG8d1-Nt*	95.7	4.3	-	-
*ATG8d2-Nt*	94.6	5.4	-	-
*ATG8e1-Nt*	95.8	4.2	-	-
*ATG8e2-Nt*	96.9	3.1	-	-
*ATG8f1-Sl*	100.0	-	-	-
*ATG8f2-Sl*	88.6	8.6	-	2.9
*ATG8f3-Sl*	86.1	11.1	2.8	-
*ATG8f4-Sl*	86.1	11.1	2.8	-

**Table 4:** Primer information for the *ATG8* gene family.

Name of Primer	Sequence	Gene ID
*ATG8a-F*	GGATGCTTTTCCACTC	JF304784
*ATG8a-R*	GTGAAGAAACAGGATACCATC	
*ATG8b-F*	GTTAAGAGCTCATTCAAGCAGGA	KR336565
*ATG8b-R*	TCCCCGAATGTGTTTTCTCCA	
*ATG8c*-F	GAGGAGGCAGGCAGAATCTT	MK189279
*ATG8c*-R	ACCCAA*ATG*TATTTTCGCCGC	
*ATG8d-F*	TGGCCAAGAGTTCTTTCAAGC	KR336567
*ATG8d-R*	CCAAGCTCAAGGAACCCAAAAG	
*ATG8e-F*	TGAACACCCCATGGAGAGGA	KR336568
*ATG8e-R*	GGAACCCAAATGTATTTTCT	
*ATG8f*-F	GGCTAAGAGCTCATTCAAGCA	NM_001247705
*ATG8f*-R	CTACAGTTCGCTCAGGACCCCGAA	

**Table 5:** qPCR primers for the *ATG8* gene family.

Name of Primer	Sequence	Reference
*ATG8a*-F	CCTGCTGATCTGACTGTGGG	[37]
*ATG8a*-R	CTGTCGGAGGAAGGATATTTTTC	
*ATG8b*-F	CAGTTGGGCAATTTGTCT*ATG*TC	
*ATG8b*-R	TTCAGGTCCCCGA*ATG*TGTT	
*ATG8c*-F	TATTCCCAACATTGACAAGAAAAAG	
*ATG8c*-R	TGACGTAGACAAACTGCCCCA	
*ATG8d*-F	CATCCGAGAGAAGTATCCCGA	
*ATG8d*-R	CAGACAACAGAGCAGCCGTG	
*ATG8e*-F	TTTGGAGAGGAGGCAGGCA	
*ATG8e*-R	CAGACATCAGAGCAGCAGTTGG	
*ATG8a*-F	CCTGCTGATCTGACTGTGGG	
*ATG8a*-R	CTGTCGGAGGAAGGATATTTTTC	

## Data Availability

Data are contained within the article.
